# Mixing of Non-Collinear Lamb Wave Pulses in Plates with Material Nonlinearity

**DOI:** 10.3390/s23020716

**Published:** 2023-01-08

**Authors:** Juan Carlos Pineda Allen, Ching Tai Ng

**Affiliations:** School of Civil, Environmental & Mining Engineering, The University of Adelaide, Adelaide 5005, Australia

**Keywords:** wave mixing, non-collinear, Lamb wave, material nonlinearity, non-planar wavefront

## Abstract

Guided waves have been extensively studied in the past few years, and more recently nonlinear guided waves have attracted significant research interest for their potential for early damage detection and material state characterization. Combined harmonic generation due to wave mixing can offer some advantages over second harmonic generation. However, studies focused on Lamb wave mixing are still very limited, and have mainly focused on collinear wave mixing and used plane wave assumption. In this paper, numerical simulations and experiments are conducted to understand the interaction of mixing non-collinear Lamb wave pulses with non-planar wavefronts. The results demonstrate that the generated secondary wave is cumulative under internal resonance conditions and the sum-frequency component of the combined harmonics is useful for characterizing material nonlinearities.

## 1. Introduction

Traditional guided wave (GW) testing is based on linear features, e.g., time-of-flight and amplitude of the scattered wave, which has been widely employed to evaluate materials and detect defects due to its outstanding capabilities, such as (a) the ability to inspect inaccessible locations, (b) the ability to inspect the entire cross-sectional area of the element, (c) excellent sensitivity, (d) cost-effectiveness, and (e) low energy-consumption. Ultrasonic techniques based on linear features are sensitive to macro scale-damage, such as gross defects or open cracks. However, they are insensitive to micro-scale damage, such as degradation or distributed micro-cracks, which are early stage damage mechanisms.

### 1.1. Nonlinear Guided Waves

An alternative solution to the aforementioned limitation is nonlinear ultrasonics [1], where the incident wave interacts with the material and generates nonlinear responses, e.g., higher harmonics and combined harmonics, due to nonlinear mechanical behavior. Harmonic generation is governed by two physical mechanisms, material nonlinearity and contact acoustic nonlinearity [2]. The material-related second-order harmonic generation has been proven efficient in detecting microstructural variations such as fatigue [3] and plasticity [4] in metallic specimens. Damage-related second harmonics generated by the contact acoustic nonlinearity phenomenon have also been studied for detecting damage, such as delamination in the composites [5] and fatigue cracks in the metals [6]. However, second harmonic generation possesses difficulty in isolating the source of nonlinearity generated by testing equipment. Contact between the inspected structure and the ultrasonic transducer may also introduce nonlinearities that can mask the material and damage-related nonlinearities. In addition, the magnitude of the second harmonic is usually very small, which is hard to measure accurately.

### 1.2. Guided Wave Mixing

To overcome these aforementioned drawbacks, some developments have been described for the wave mixing method in the last few years, which makes use of two ultrasonic waves at different single central frequencies [7]. The wave mixing approach can be categorized as collinear [8] and non-collinear [9] depending on the angle between the incident (primary) wave that generate the resonant (secondary) wave at combined harmonics, and offer advantages in selecting wave modes, excitation frequencies, and wave propagating directions.

Croxford et al. [9] employed bulk waves to characterize material nonlinearity due to plasticity and fatigue deterioration using the non-collinear approach. Using experimental results with aluminum alloy, material nonlinearity was correlated to plastic deformation. A nonlinear acoustic parameter for collinear bulk wave mixing related to plastic deformation was introduced and validated numerically and experimentally [10]. Experimental studies demonstrated that the collinear method could measure localized plastic deformation [11]. Jingpin et al. [12] determined the presence of fatigue cracks using bulk shear waves and the non-collinear method. Co-directional Lamb wave mixing in steel plates was used to investigate micro-crack damage [13] and localized creep [14]. Wave-mixing was also demonstrated to be effective with an edge wave [15].

### 1.3. Lamb Wave Mixing

Ultrasonic Lamb waves offer significant advantages for the evaluation of structures, such as the ability to inspect inaccessible locations, multimodality, flexibility in wave mode selection, ability to inspect multilayered or submerged structures, etc. Hasanian et al. [16] conducted comprehensive vector analyses for both collinear and non-collinear methods using Lamb waves. However, they assumed that the guided wave was a plane wave to explore the possible wave-mode combination triplets. Arbitrary angles between primary waves and secondary angle were studied, and internal resonant conditions were also assessed for wave mixing [17]. Ishii et al. [18] theoretically analyzed nonlinear wave propagation in an isotropic and homogenous plate to elucidate the non-collinear interaction of monochromatic plane waves employing infinite beam widths. They also conducted finite element analyses considering finite beam width and time durations to provide a further understanding of the scattered wave generation. Li et al. [19] studied wave-mixing responses induced by co-directional Lamb waves and predicted the generation of second- and third-order combined harmonics.

Collinear wave mixing has the advantage that the wave mixing zone is larger than non-collinear wave mixing, but this makes it less suitable for inspecting localized regions. Counter-propagating wave mixing offers an advantage in evaluating localized regions in the structures [20]. However, non-collinear wave mixing offers more flexibility in the selection of the primary and secondary wave modes [17]. Analytical solutions for plane waves were developed for Lamb wave mixing but they are insufficient in capturing the real testing conditions, such as the non-planar wavefront and the influence of finite ultrasonic beam width on wave interaction. For practical applications, they need to take into account these practical factors in Lamb wave mixing. Gaining physical insight into the Lamb wave mixing phenomenon will expand the already recognized benefits and further advance wave mixing methods.

The main objective of this paper is to investigate the feasibility and capability of combined harmonic measurements with non-collinear Lamb wave mixing under practical conditions. It focuses on gaining insight into the physical phenomena of secondary symmetric Lamb wave generation at a combined sum frequency when two fundamental modes of antisymmetric Lamb waves $\left(A_{0}\right)$, which have finite beam width, non-planar wavefront, and finite time duration, interact with each other over a localized region under resonance conditions. The interaction of the two $A_{0}$ Lamb waves is expected to generate a secondary fundamental mode of symmetric Lamb wave $\left(S_{0}\right)$, and this will be studied in detail under a practical situation. The cumulative nature of the secondary Lamb wave at a combined sum frequency is further investigated, as this feature has proven to be a useful indication of material nonlinearity, fatigue, and plasticity.

## 2. Theoretical Background

### 2.1. Internal Resonance Criteria for Harmonics Generation

Second-order harmonics generated due to interaction between two primary waves are cumulative when internal resonance criteria are satisfied [21,22,23]. The two internal resonance criteria are (i) non-zero power flux from the primary to the secondary mode and (ii) phase matching. The non-zero power flux condition in Equation (1) guarantees that power is transmitted through the surface and the volume of the plate due to the primary wave, while the phase matching condition guarantees a sustained power flux from the primary to the secondary waves.

(1) $$f_{n}^{vol}+f_{n}^{surf}\neq 0$$

where the terms $f_{n}^{vol}$ and $f_{n}^{surf}$ are the driving forces transmitting power from the primary waves to the secondary wave through the volume and the surface, respectively. 

**Figure 1:**
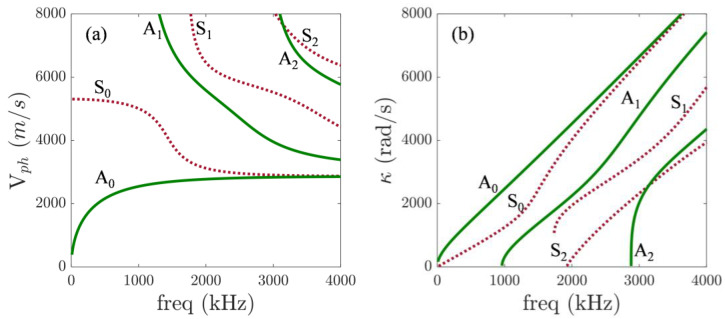
(**a**) Phase velocity and (**b**) wavenumber dispersion curves for a 1.60 mm thick aluminum plate.

The phase matching condition in Equation (2), also known as synchronism, requires knowledge of the dispersion characteristics represented in the dispersion curves of Figure 1. In this study, dispersion curves were calculated using DISPERSE, under the assumption that the wavefront is an infinite plane and normal to the direction of wave propagation. Waves can propagate in different directions; thus, when seeking triplets that are phase matched, wave vectors containing wave number and wave direction information are used. The wavenumber obtained from the interaction between the primary wave must correspond to a propagating mode, and the phase velocity is then calculated with the relationship $c_{ph}=2\pi f/\kappa$, where $c_{ph}$ is the phase velocity. $\kappa_{a}$, $\kappa_{b}$, and $\kappa_{n}$ are the wavenumbers for primary waves a, b, and secondary wave n.

(2) $$\kappa_{a}\pm \kappa_{b}=\kappa_{n}$$

Most of the work developed to date is derived from plane waves with infinite beam widths, aiming to analytically understand the basis of wave mixing phenomena; however, they are not fully practical for real applications. Therefore, finite element simulations and experiments are needed to further understand Lamb wave mixing in a practical situation.

### 2.2. Modelling of Material Nonlinearity

In the finite element simulations, the material nonlinearities and field equations can be modelled by considering the third-order terms of Murnaghan’s strain energy function [24,25,26]. Assuming the reference configuration is ***X*** and the current configuration of material is ***x***, the displacement vector ***u*** is

(3) u=x−X

and the displacement gradient tensor ***F*** is

(4) $$F=\frac{\partial x}{\partial X}=I+H$$

where H=∂u/∂X is the displacement gradient and ***I*** is the identity tensor. For the Isotropic medium, the second Piola–Kirchhoff stress can be expressed in terms of Murnaghan’s strain energy function *W*:

(5) $$T_{pk2}=\frac{\partial W\left(E\right)}{\partial E}$$

where $E=\left(H+H^{T}+H^{T}H\right)$ is the Green–Lagrange strain tensor. Using the principal invariants *i*_1_, *i*_2_, and *i*_3_, the Murnaghan’s strain energy function can be represented as

(6) $$W\left(E\right)=\frac{1}{2}\left(\lambda +2\mu \right)i_{1}^{2}+\frac{1}{3}\left(l+2m\right)i_{1}^{3}-2\mu i_{2}-2mi_{1}i_{2}+ni_{3}$$

where λ and μ are the classical Lam$\overset{´}{e}$ elastic constants and *l*, *m*, and *n* are the third-order elastic constants. $i_{1}=tr\left(E\right)$, $i_{2}=\left[i_{1}^{2}d-tr{\left(E\right)}^{2}\right]/2$, and $i_{3}=\det\left(E\right)$. Therefore, the Cauchy stress can be expressed in terms of the second Piola–Kirchhoff stress, $T_{pk2}$, and deformation gradient, F**,** as

(7) $$\sigma =J^{-1}F\frac{\partial W\left(E\right)}{\partial E}F^{T}$$

where $J^{-1}$ is the Jacobian determinant of the deformation gradient, F. Using Equation (7), the constitutive behaviors of materials can be used to model the weak material nonlinearity using the VUMAT subroutine of ABAQUS/Explicit.

## 3. Finite Element Simulation

A three-dimensional (3D) finite element model developed using ABAQUS/Explicit was used to investigate non-collinear Lamb wave mixing. The model is a 6061-T6 aluminum plate. The material properties of the 6061-T6 are listed in Table 1. The dimensions of the plate are 270 mm × 320 mm × 1.60 mm. The in-plane dimensions of the element used in the finite element model are 0.25 mm × 0.25 mm. There are seven layers of elements in the thickness direction of the plate and each element is 0.228 mm thick. Approximately 20 elements/wavelength is the recommended spatial resolution for representing the propagation of a Lamb wave [27]. The increment time step was automatically controlled by ABAQUS/Explicit. Eight-noded brick elements, C3D8R, with each node containing three translational degrees-of-freedom, and reduced integration were used. Mechanical constitutive behavior based on the nonlinear strain energy function of Murnaghan was modelled in ABAQUS through a VUMAT subroutine [28].

**Figure 2:**
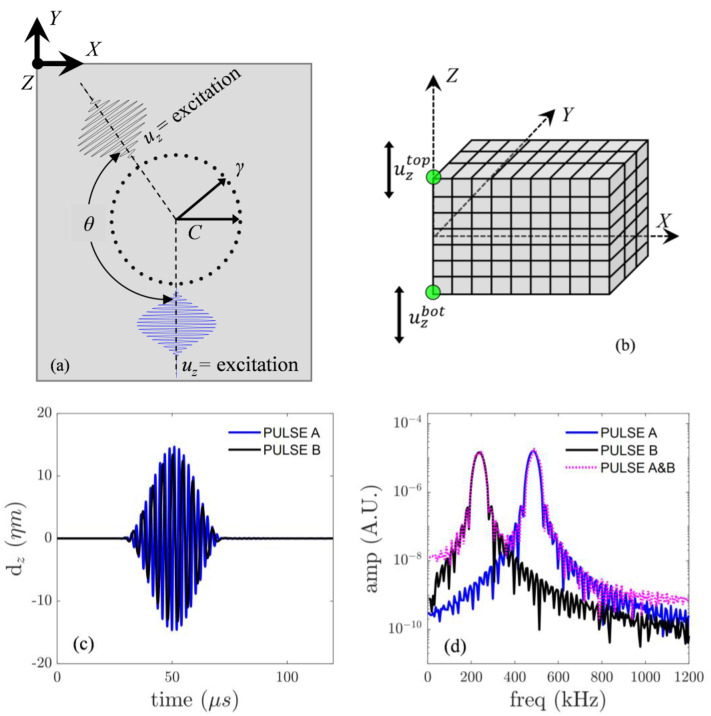
(**a**) Schematic diagram of the non-collinear FE model, (**b**) top and bottom nodal displacements, (**c**) time-domain, and (**d**) frequency spectra of out-of-plane displacement of incident waves at point *C*.

To contemplate practical applications, primary Lamb wave excitations consisted of tone burst pulses applied through nodal displacement in the z direction to the nodes covered by the assumed ultrasonic transducer, as indicated in Figure 2a. The interaction angle between the primary waves was defined by θ. The maximum applied displacement was 20 ηm. All the remaining edges and boundaries of the plate were stress-free. An appropriate time delay was applied to the pulse excitation of the Lamb wave with faster group velocity to ensure both wave pulses arrived simultaneously in the region of interest. Moreover, the distance between each excitation source and the mixing zone, the center of which is defined as the point C, were equal to each other. The primary waves were expected to interact at a finite region; hence, they is not limited at the point C only.

To extract the scattered wave field, a subtraction technique [16] was employed. Three different finite element simulations were carried out separately. In this approach, all three simulations were performed with the identical finite element model, but the generated wave is different. Three different generated waves were considered: (i) primary waves were generated simultaneously $\left(T_{A\&B}\right)$, (ii) only primary wave, A $\left(T_{A}\right)$, was generated, and (iii) only primary wave, B $\left(T_{B}\right)$, was generated. The scattered wave field can be extracted by

(8) $$T_{sca}=T_{A\&B}-T_{A}-T_{B}$$

The out-of-plane nodal displacement at the top and bottom surfaces of the plate, as shown in Figure 2b, was obtained from each simulation. Displacement was obtained from nodes located along a circle of 30 mm radius for a range of γ angles from 5° to 360° in steps of 5°. The secondary symmetric Lamb wave was obtained by Equation (9)

(9) $$u_{sca}=\frac{1}{2}\times \left[u_{sca}^{top}-u_{sca}^{bot}\right]$$

where

(10) $$u_{sca}^{top}=u_{z_{A\&B}}^{top}-u_{z_{A}}^{top}-u_{z_{B}}^{top}$$

(11) $$u_{sca}^{bot}=u_{z_{A\&B}}^{bot}-u_{z_{A}}^{bot}-u_{z_{B}}^{bot}$$

In Equation (10), the first summand corresponds to the out-of-plane nodal displacement at the top surface of the plate when primary waves A and B are excited simultaneously, the subtrahends correspond to the out-of-plane nodal displacement at the top surface of the plate when primary wave A only and primary wave B only are excited separately. A similar interpretation is applied for Equation (11), although these values are obtained from the bottom surface of the plate.

Similarly, Equation (12) can be used to obtain antisymmetric incident Lamb wave

(12) $$u_{inc}^{A}=\frac{1}{2}\times \left[u_{inc}^{top}+u_{inc}^{bot}\right]$$

where

(13) $$u_{inc}^{top}=u_{z_{A}}^{top}$$

(14) $$u_{inc}^{bot}=u_{z_{A}}^{bot}$$

for simulation A. Correspondingly for simulation B,

(15) $$u_{inc}^{B}=\frac{1}{2}\times \left[u_{inc}^{top}+u_{inc}^{bot}\right]$$

where

(16) $$u_{inc}^{top}=u_{z_{B}}^{top}$$

(17) $$u_{inc}^{bot}=u_{z_{B}}^{bot}$$

Excitation frequencies were selected so that the internal resonance conditions derived from the plane wave assumption were theoretically satisfied. Additionally, wave mode selection is a determining factor for practical applications of nondestructive testing, and unwanted higher-order wave modes can be avoided by employing fundamental modes. Hence, a fundamental mode below the cut-off frequency was employed in this study. In particular, we are interested in primary $A_{0}$ waves that are expected to generate a secondary cumulative wave, $S_{0}$, propagating mode [23], where the nonzero power flux condition for non-collinear wave-mixing is satisfied by choosing the appropriate wave modes. The synchronism condition was employed to find dispersion relations based on the frequency and direction. The triplet selected was: $f_{a}=484\;\mathrm{kHz}$ ($c_{ph}=2141.9\;m/s$, $\kappa_{a}=1419.8\;\mathrm{rad}/m$), $f_{b}=230\;\mathrm{kHz}$ $(c_{ph}=1658.9\;m/s$, $\kappa_{b}=871.1\;\mathrm{rad}/m)$, and $f_{sca}=714\;\mathrm{kHz}\;(c_{ph}=5186.5\;m/s$, $\kappa_{sca}=864.9\;\mathrm{rad}/m$). The interaction angle θ was 145° and the expected direction, γ, of the resonant wave was 55°. The number of cycles was 31 and 15 for pulse A and B, respectively.

$A_{0}$ wave fields, $T_{A}$ and $T_{B}$, at point C are shown in Figure 2c. Maximum interaction occurs, with the maximum displacement of both incident pulses occurring just after 50 µs. Figure 2d shows the frequency spectra of the $A_{0}$ component for the three simulation cases, which correspond to wavefields $T_{A}$, $T_{B}$, and $T_{A\&B}$. The displayed frequency spectra show the excitation frequencies acting separately as well as concurrently. From this stage, the subtraction technique is required to extract the $S_{0}$ component using Equations (8) and (9).

**Figure 3:**
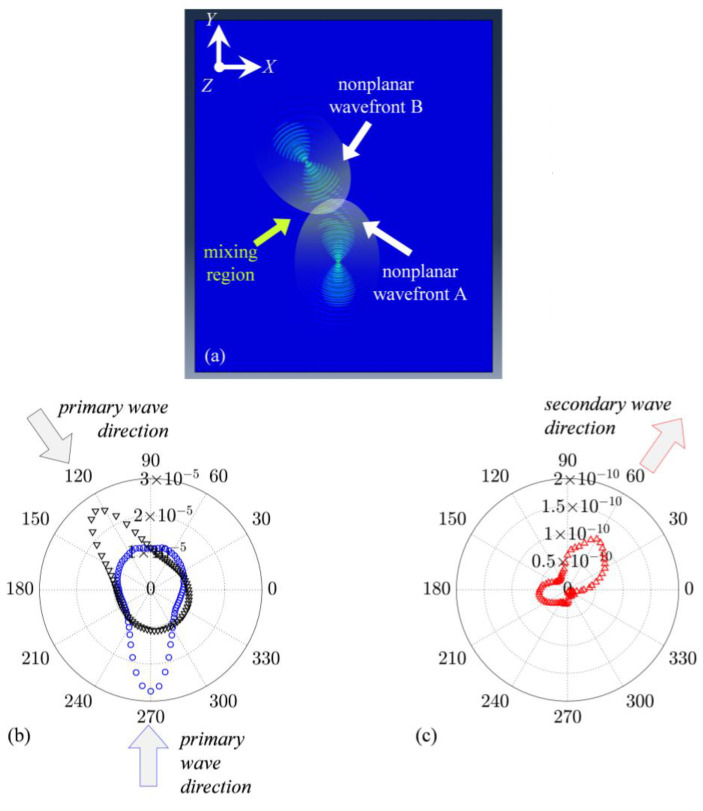
(**a**) Out-of-plane displacement at the top surface when *A* and *B* occur simultaneously, (**b**) amplitude of *A_0_* component of simulations *A* at *f_a_* and *B* at *f_b_*, (**c**) amplitude of *S_0_* component at *f_sca_*.

The non-planar primary Lamb waves are expected to propagate over a region that is not limited to point C. A snapshot of the displacement in the z direction of the top surface of the plate is shown in Figure 3a. The displacement over a region of the plate must contain frequency components at excitation frequencies. The $A_{0}$. primary wave A and primary B were obtained by summing the out-of-plane nodal displacement at the top and bottom surface of the plate and their amplitude spectra at the corresponding $f_{a}$ and $f_{b}$ were calculated. Figure 3b shows the polar plots of the directivity pattern of the incident wave amplitudes, which were obtained from the magnitudes at the excitation frequencies of the amplitude spectra.

The interaction between two antisymmetric propagating Lamb waves is expected to produce a symmetric Lamb wave at combined harmonics due to the wave mixing effect. Interaction occurs over a mixing region, as shown in Figure 3a; hence, the secondary symmetric mode of the Lamb wave due to the local interaction effect is expected over this region. The combined harmonic is expected to be cumulative along the scattered wave angle in view of the resonance condition. $S_{0}$ was obtained using Equation (9), and the amplitude spectra at the corresponding $f_{sca}$ was calculated, as shown in Figure 3c.

Theory based on plane wave assumption can predict the direction of the scattered wave, but it has limitations in that it is not fully practical for real applications. However, with numerical simulations, we can understand how wave mixing phenomena occur when primary Lam. waves with finite beam width and non-planar wavefront interact with each other. It can be seen from Figure 3c that the directivity pattern of the secondary, $S_{0}$, at the combined sum frequency, $f_{sca}$, shows that maximum amplitude occurs towards the resonant angle. Some deviation could be expected given that dispersion curves from Figure 1 were calculated based on the plane wave assumption. Cumulative nature of the secondary wave at the combined frequency is then evaluated at the expected resonant direction (γ=55°). From the frequency domain data of the extracted $S_{0}$ component of the scattered Lamb wave, the area under the Fourier amplitude spectrum curve at $f_{sca}=714\;\mathrm{kHz}\;$was calculated using the trapezoidal rule. This calculated area is the numerator $A_{3}$. Similarly, the areas under the Fourier amplitude spectra curves at $f_{a}=484\;\mathrm{kHz}$ and $f_{b}=230\;\mathrm{kHz}$ were calculated from the frequency domain data of the $A_{0}$ component of the incident pulses; the product of these areas is then $A_{2}A_{1}$. The nonlinear parameter is defined as:

(18) $$\beta =\frac{A_{3}}{A_{2}A_{1}}$$

**Figure 4:**
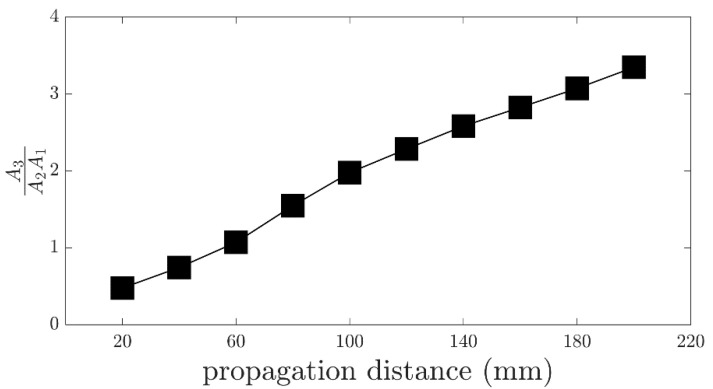
Cumulative behavior of the secondary wave.

The nonlinear parameter, β, was calculated along ten points every 20 mm in the γ=55° direction. The results are shown in Figure 4, showing a linearly increasing trend with cumulative nature due to material nonlinearity.

## 4. Experimental Study

**Figure 5:**
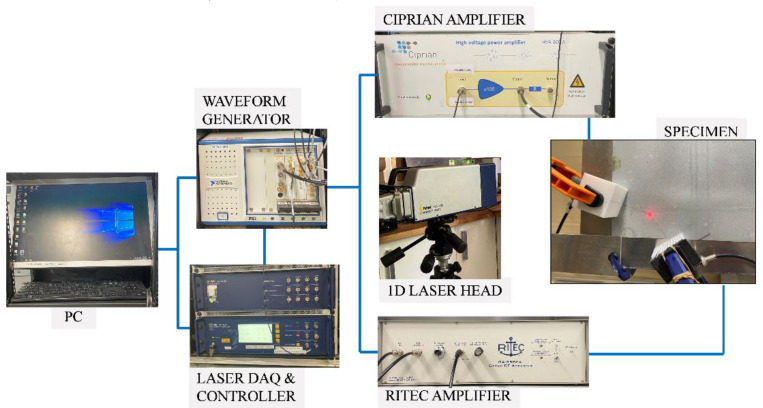
Experimental setup.

A 300 mm × 300 mm × 1.60 mm 6061-T6 aluminum plate was used in an experiment to observe the wave mixing responses of two primary non-collinear Lamb waves due to their mutual interaction under resonance conditions. Two wedge transducers designed for generating antisymmetric propagating mode Lamb waves were used. The wedge transducer consisted of a Teflon wedge and a longitudinal wave transducer. Using Snell’s law, the oblique angles of the wedges were calculated as $\theta_{a}=38{}^{\circ}$ ($f_{a}\;$= 484 kHz) and $\theta_{b}=53{}^{\circ}\;$($f_{b}$ = 230 kHz). Two PC-controlled NI PXI-5412 waveform generators were used to generate two independent signals. Both signals consisted of sinusoidal tone-burst waves modulated by a Hann window. Signals were amplified by two separate amplifiers. Transducer-wedge interface and wedge-specimen interface were coupled with light motor oil and clamped to the surface of the plate. Given that the wedges had different Snell’s angles, the distance between the transducer and the plate was also different because of the manufacturing process of the wedges. Thus, preliminary studies were conducted so that both actuated waves had approximately similar maximum amplitude. Pulse A was a 31-cycle tone burst pulse with a central frequency of 484 kHz, amplified to 100 Vpp, and Pulse B was a 15-cycle tone burst pulse with a central frequency of 230 kHz, amplified to 120 Vpp. Since the pulse with $f_{a}$ frequency propagates faster than the pulse with $f_{b}$ frequency, an appropriate time delay was applied during the excitation signal generation; the distance between the wedge transducers and measurement point was kept constant. A schematic diagram is shown in Figure 5.

## 5. Discussion

Tests to explore the generation of the antisymmetric Lamb wave were conducted first to confirm the successful generation of the primary waves. To confirm this, each transducer was actuated separately and the out-of-plane displacements at different locations were measured using a 1D scan measured by a Polytec PSV-400-M2-20 scanning laser vibrometer (SLV). Group velocity was calculated using the time-of-flight of the signal envelope obtained from the Hilbert transform and the distance between consecutive measurement points. The group velocity was $V_{gr}=3032\;m/s$ for pulse A and $V_{gr}=2701\;m/s$ for pulse B, which were in good agreement with the analytical values from the dispersion curve corresponding to the $A_{0}$ wave ($V_{gr}=3036\;m/s$ and $V_{gr}=2697\;m/s$). To increase the optical backscatter reflection of the laser beam, a reflective paint layer was applied to the surface of the specimen. The measured signals were averaged 500 times to increase the quality of the measurements and to reduce potential errors arising from measurement noise.

**Figure 6:**
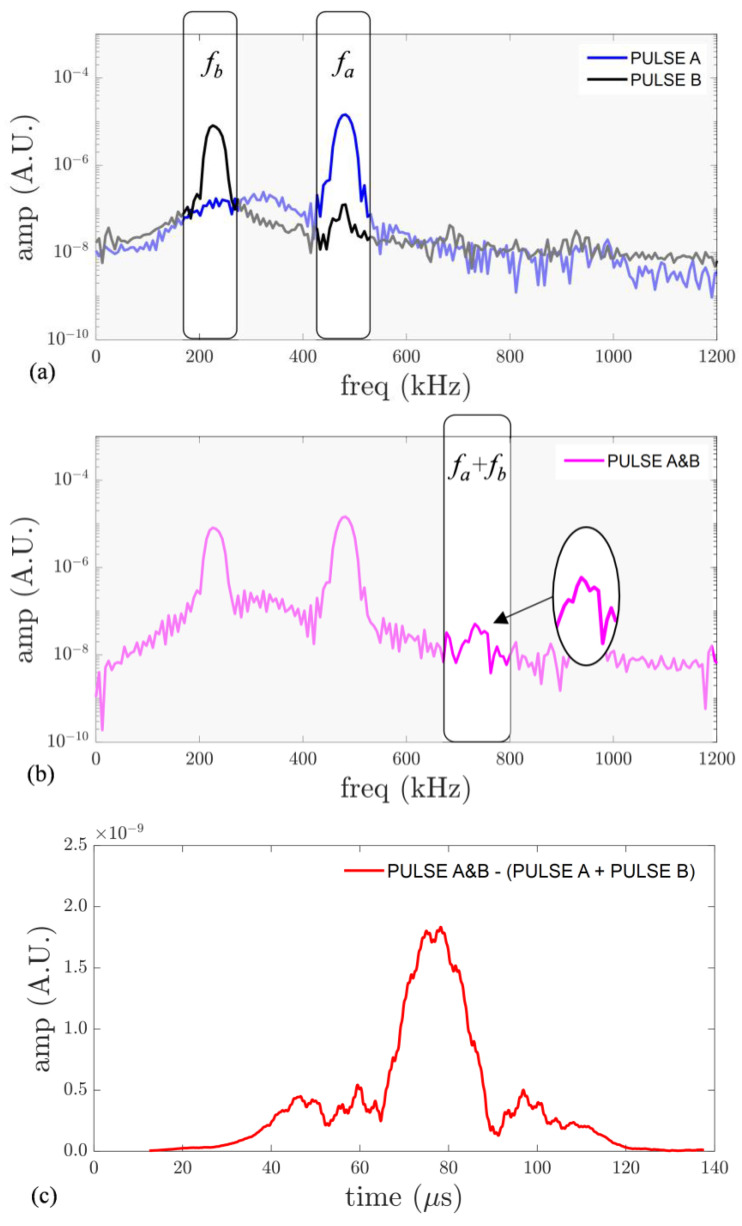

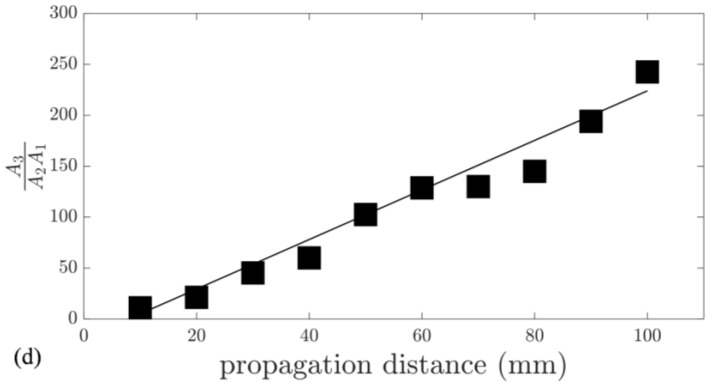
(**a**) Frequency spectra when pulses are excited separately, (**b**) frequency spectra when pulses are excited simultaneously, (**c**) extracted nonlinear wavefield, and (**d**) experimental cumulative behavior of the secondary wave.

Pulses A and B were excited simultaneously to study their mutual interaction. Additionally, pulses A and B were excited separately to extract the combined harmonic that only occurs when Lamb waves interact simultaneously. The frequency content of the out-of-plane displacement in the mixing zone is shown in Figure 6a when the pulses were excited separately and Figure 6b when the pulses were excited simultaneously. Additional frequency components due to mutual interaction were observed at two combined frequencies, namely $f_{a}+f_{b}$ and $2f_{a}+f_{b}$. They did not appear when pulses were excited separately. In this paper, we aim to report the secondary wave at the combined sum frequency, $f_{a}+f_{b}$, as an indication of material nonlinearity. The extracted nonlinear wavefield, which is at $f_{a}+f_{b}=730\;\mathrm{kHz}$, is also shown in Figure 6c. In this study, the presence of the combined harmonic should not be polluted by equipment nonlinearity given that the signals are amplified by two different power amplifiers. Moreover, the use of two different transducers for actuation and a non-contact laser for measurement should not cause combined harmonic generation. Nonetheless, the amplitude of the combined harmonic is still relatively small; hence, there could certainly be some level of measurement error. To assess the cumulative nature of the generated secondary pulse, a different set of measurements was conducted along a line of ten sensing points in 10 mm intervals at the expected direction of the resonant wave, γ=55°. Then, the amplitude spectrum of the secondary wave was normalized by the product of the amplitude spectrum of the primary waves and plotted as a function of propagation distance, as shown in Figure 6d. This linear increase indicates that the secondary wave can grow cumulatively [21], thereby validating its cumulative nature with propagation distance. As such, in plate-like structures, this practical phenomenon has proven to be practical and useful for characterizing weak material nonlinearity, plasticity, and fatigue [9].

## 6. Conclusions

This study has investigated the interaction of mixing non-collinear Lamb wave pulses with a non-planar wavefront in an isotropic plate. Practical conditions of finite beam width, finite pulse duration, and finite interaction region have been considered in this study. A finite element model with weak material nonlinearity, modelled using third-order terms of Murnaghan’s strain energy function, has been employed to demonstrate the generation of the combined harmonic generation when two incident Lamb waves with nonplanar wavefronts interact with each other under the resonant condition. An experiment using two separate transducers with two separate amplifiers has been conducted to demonstrate that when two incident Lamb waves with non-planar wavefronts and finite time duration interact with each other under resonant conditions, the combined harmonic has a cumulative nature due to material nonlinearity. Given the non-planar wavefront generated by the incident pulse, a physical insight indicates that combined harmonic generation takes place due to the local interaction effect over the mixing region, and that the magnitude distribution of this local effect is consistent with the internal resonance criteria. Moreover, the use of $A_{0}$ Lamb waves introduces the potential for advancing wave mixing damage detection techniques, such as debonding, delamination, or impact, which have not been fully investigated in the literature.

## Figures and Tables

**Table 1:** Material properties used in the finite element model.

Density	*l* (GPa)	*m* (GPa)	*n* (GPa)	*λ* (GPa)	*μ* (GPa)
2704	−281.50	−339	−416	54.3	27.3

## Data Availability

Not applicable.
